# Characterisation of Early Microbial Colonisers within the Spiral Colon of Pre- and Post-Natal Piglets

**DOI:** 10.3390/life11040312

**Published:** 2021-04-02

**Authors:** Tanya L. Nowland, Roy N. Kirkwood, Valeria A. Torok, Kate J. Plush, Mary D. Barton

**Affiliations:** 1 School of Animal and Veterinary Sciences, The University of Adelaide, Roseworthy, SA 5371, Australia; roy.kirkwood@adelaide.eu.au (R.N.K.); valeria.torok@sa.gov.au (V.A.T.); 2 South Australian Research and Development Institute, Food Sciences, Urrbrae, SA 5064, Australia; 3 SunPork Group, Murarrie, QLD 4172, Australia; kate.plush@sunporkfarms.com.au; 4 School of Pharmacy and Medical Sciences, University of South Australia, Adelaide, SA 5000, Australia; mary.barton@unisa.edu.au

**Keywords:** microbiota, stillborn piglets, bacterial abundance, archaea

## Abstract

Initial enteric microbial colonisation influences animal health and disease, hence an understanding of the first microbial colonisers within the piglet is important. The spiral colon of piglets that were stillborn (*n* = 20), born-alive (*n* = 10), and born alive and had sucked (*n* = 9) were collected from 28 sows to investigate whether initial microbial colonisation occurs pre- or post-partum and how it develops during the first 24 h post-partum. To examine this, DNA was extracted and 16S rRNA amplicon analysis was performed to allow analysis of microbial communities. The results indicate that microbial colonisation of the spiral colon had occurred in stillborn pigs, suggesting microbial exposure prior to birth. Alpha diversity metrics indicated that the number of taxa and community richness were higher in piglets that sucked (*p* < 0.001) and community evenness was lower in stillborns in comparison to born-alive (*p* < 0.001) but was not affected by colostrum consumption (*p* < 0.001). Additionally, when compared with stillborn piglets, the bacteria colonising the spiral colon during the first 24 h post-partum included the potentially pathogenic bacteria *Escherichia coli*, *Clostridium perfringens* and *Clostridium celatum*, and potentially beneficial bacteria *Lactobacillus reutueri* and *Faecalibacterium prausnitzii*. The relative presence of Archaea was high in stillborn piglets but decreased with post-natal environmental exposure. It is evident that stillborn piglets have bacteria present within their spiral colon, however further studies are needed in order to determine the time at which colonisation is initiated and the mechanisms determining how colonisation occurs. Additionally, as expected, the immediate post-natal environment largely influences the microorganisms colonising, while colostrum consumption further contributes to the microbial community enrichment.

## 1. Background

The first colonisers within the gastrointestinal tract (GIT) play a determinant role in the health of the host [1,2], therefore it is important to understand when colonisation occurs, and which are the main bacteria involved in early life colonisation. Advances in sequencing technologies have allowed for new observations in understanding the timing of initial microbial colonisation, as neonates were previously thought to be sterile until birth. Research in humans [3], mice [4] and rhesus macaques [5] indicate possible colonisation by bacteria *in utero*, with studies demonstrating the presence of bacteria in the amniotic fluid, placenta and meconium of healthy neonates. Even with these findings it is still a topic of debate with some studies refuting the likelihood of in utero colonisation [6,7]. Additionally, the fact that it is not clear how the microbes colonise these surfaces furthers this notion. Some studies suggest that the maternal oral or intestinal microbes may be selectively transported to the fetal-placental interface as has been shown previously for the transport of bacteria to mammary tissue in humans [3,8,9]. Additionally, studies in humans have shown that fetuses ingest large amounts of amniotic fluid in late gestation which may aid GIT colonisation [10,11]. Few studies have investigated the bacteria present in the GIT within the first days of life in piglets [12,13]. Early microbial colonisation via the vagina, nipple surface and milk have been documented extensively in humans [14,15] with some work reported in pigs [16]. To our knowledge, no studies have documented microbial colonisation of piglets at any timepoint throughout gestation or in piglets that have not sucked. It is important to identify the first microbes colonising the GIT as it will allow for the planning of nutritional interventions in sows or newborn piglets to increase their survivability, feed efficiency and growth. Evidence suggests that pre and probiotics fed to the sow are effective in altering the microbiota of piglets during lactation [17,18] and so, therefore, the potential to utilise these interventions during gestation to foster the development of an advantageous intestinal microbiota in piglets is of interest. Therefore, microbial colonisation just prior to birth and during the immediate post-natal period was studied using the spiral colon of stillborn piglets and piglets prior to sucking. This study aimed to determine GIT colonisation before and immediately after birth in piglets that had or had not sucked. It was hypothesised that (1) passive transfer of microbes just prior to birth would occur and, therefore, microbes would be present in the spiral colon of stillborn piglets and (2) the composition, abundance and diversity of communities colonising the spiral colon would increase with birth, environmental exposure and the consumption of colostrum.

## 2. Materials and Methods

### 2.1. Animals and Experimental Procedures

All procedures were conducted at the University of Adelaide Roseworthy piggery, South Australia, with the approval of the University of Adelaide’s Animal Ethics Committee (AEC number: S-2018-092). A total of 39 Large White x Landrace piglets born to 28 sows (parities 3.84 ± 0.34) were employed in this study over a series of two batches. All sows were group housed throughout gestation and did not receive antibiotics. They received 2.5 kg/d of commercial gestation diet (12.85 MJ DE/kg) throughout gestation. Upon entry into farrowing accommodation sows received a commercial lactation diet (14 MJ DE/kg) at 2.5 kg/d until farrowing, thereafter the feeding level was gradually increased until it reached 7 to 8 kg by day 7 of lactation. All sows had *ad libitum* access to water. Farrowing accommodation consisted of farrowing crates (1.7 m × 2.4 m) located in rooms that were climate controlled and had fully slatted plastic flooring. Sows were moved into farrowing accommodation five days prior to their expected due date. Sows farrowed naturally and were monitored during staffed hours from 8 a.m. to 3 p.m. On average, the sows gave birth to a total of 14.1 ± 0.6 piglets per litter, with an average gestation length of 115.2 ± 0.3 days. When sows were separated into Born-Alive, Sucked and Stillborn groups, no treatment differences existed for total born or gestation length. Of the 39 piglets employed, they consisted of 20 stage II stillborn piglets (stillborn), as defined previously [19], (9 female, 11 male), 10 euthanised or recently crushed 0–1 day old born-alive piglets that had not sucked (born-alive; 5 female, 5 male) and 9 euthanised or recently crushed 0–1 day old live-born but non-viable piglets that had sucked (sucked; 4 female, 5 male). Piglets were classified as stillborn if they had intact cartilaginous tips on their hooves and they had not taken a breath, as indicated by a lung float test. Piglets deemed as non-viable by the farm staff due to piglet size, splay legs or viability were euthanised by blunt force trauma to the skull with immediate exsanguination. The presence or absence of milk in the stomach was used as an indication of whether liveborn piglets had sucked or not. The average weights for piglets in the stillborn, born-alive and sucked groups were 1.03 ± 0.06 kg, 0.75 ± 0.04 kg and 1.14 ± 0.12 kg, respectively. Piglets were placed on ice immediately and transported to the laboratory for dissection within one hour of parturition or post-natal death. Piglets were weighed and cleaned with 70% alcohol and a midline incision was made from the sternum to the pubis. The spiral colon was lifted with sterile forceps and an incision was made at either end to remove it. The spiral colon was used as it was an easy to identify area within the piglet that was in the lower region of the GIT. Therefore, if ingestion of fluid during parturition occurred, it would not have affected the results. Once dissected, the spiral colon segment was placed into a sterile tube and stored at −80 °C until DNA extraction. Utensils were changed between each incision in order to reduce the likelihood of contamination.

### 2.2. Extraction of DNA and 16S rRNA Amplicon Sequencing

Total nucleic acid was extracted and purified from freeze dried piglet spiral colon samples by a modification of a South Australian Research and Development Institute proprietary method [20,21,22]. Approximately 0.9 gm of freeze-dried spiral colon was added to 10 mL of extraction buffer (1.3 M guanidine thiocyanate, 1.5 M NaCl_2_, 30 mM Tris-HCl, 65 mM phosphate buffer, 3.4% (w/v) sarkosyl and 1.7% (w/v) polyvinylpolypyrrolidone) and incubated for 1 h at 70 °C prior to proceeding with the proprietary extraction method.

PCR amplification and sequencing of the V3-V4 region of the 16S rRNA gene was done by the Australian Genome Research Facility (AGRF) Melbourne node. The V3-V4 region was PCR amplified over 29 cycles using forward primer 341-F (CCTAYGGGRBGCASCAG) and reverse primer 806-R (GGACTACNNGGGTATCTAAT). Amplicon sequencing was done on the illumina MiSeq platform (San Diego, CA, USA) with 2 × 300 bp paired-end chemistry. Both positive and negative controls were used on every plate processed by AGRF. The positive control used was ZymoBIOMICS Microbial Community DNA Standard II (Log Distribution). The obtained reads are available under the accession number PRJNA677620 of the Sequence Read Archive of the National Centre for Biotechnology Information. For bioinformatic analysis of raw sequence data performed by AGRF, the paired-end reads were assembled by aligning the forward and reverse reads using PEAR v0.9.5 [23]. Primers were identified and trimmed. All trimmed sequences were processed using Quantitative Insights into Microbial Ecology (QIIME 1.8.4) [24] and USEARCH v8.0.1623 [25,26] software and the UPARSE pipeline [27]. Using USEARCH tools, sequences were quality filtered, and full-length duplicate sequences were removed and sorted by abundance. Singletons or unique reads in the dataset were discarded. Additionally, chimeric sequences were clustered and removed using “rdp_gold” database as the reference. To obtain the number of reads in each operational taxonomic unit (OTU), reads were mapped back to OTUs with a minimum identity of 97%. Using QIIME, taxonomy was assigned with Greengenes database (version 13.8, August 2013) [28]. All sequences corresponding to mitochondria and chloroplasts were removed.

### 2.3. Statistical Analysis

The alpha diversity metrics, Shannon diversity (H’) index, Pielou’s evenness (J’) and number of taxa (S), were calculated using DIVERSE (PRIMER6 PRIMER-E Ltd., Ivybridge, UK). Normality was tested within RStudio software (Version 1.1.456, Boston, MA, USA) using the Shapiro–Wilk test. Those alpha diversity metrics that were normally distributed were analysed using an analysis of variance (ANOVA) and those not normally distributed were analysed using the Kruskal–Wallis test, with corrections for multiple tests using false discovery rate (FDR) and a P-value threshold of 0.05. The fixed effects included in the model were group (stillborn, born-alive and sucked) and gender. The gender and the gender x group interaction were not significant (*p* > 0.05) so were removed from the final model.

Multivariate statistical techniques (PRIMER6, PRIMER-E Ltd., Ivybridge, UK) were used to analyse the spiral colon 16S rRNA bacterial taxonomic data. For phyla, family and genus levels species accumulation plots were generated on standardised by total and fourth root transformed data. Plots were generated on permuted (max 999) data. Indices investigated were: S, Chao, Jacknife, Bootstrap, Michaelis Menton and Ugland-Gray-Elligsen. Similarities among colonic bacterial communities of piglets from the 16S rRNA data metrics were analysed using Bray–Curtis measures of similarity [29] following standardisation by total and fourth-root transformation. One-way analysis of similarity (ANOSIM) [30] on the Bray–Curtis similarity data was used to test if there were significant differences among colonic bacterial communities for piglets that were stillborn, born-alive or had sucked. If the global R statistic was significant (*p* ≤ 0.05), then the significance of pairwise R statistics were investigated further. The R statistic value describes the extent of similarity among or between groups, with values close to unity (1) indicating that groups are entirely separate and a zero-value indicating that there is no difference among or between groups. To determine which individual bacterial taxa contributed most to the overall dissimilarity between significant pair-wise comparisons, similarity percentages (SIMPER) [30] analyses were done on the Bray–Curtis dissimilarity data. The percentage contributions (%) of significant taxa (average dissimilarity/standard deviation > 1) to the average dissimilarities were calculated. Non-metric multidimensional scaling (nMDS) [31,32] on Bray–Curtis similarity data was done to graphically illustrate relationships among the groups.

## 3. Results

Across all 39 samples, the total number of sequenced reads were 8,772,894, of which 4,648,730 reads were retained after quality control with an average of 119,198 sequenced reads per spiral colon sample. The number of reads clustered into OTUs were 4281. Species accumulation curves and richness indices of the bacterial communities in the spiral colon of piglets in the stillborn, born-alive and born-alive and sucked groups were performed. The calculated species accumulation indices reached an asymptote after ~9 samples, indicating that this number of samples allowed for the detection of most bacterial genera present and that the number of replicates per treatment were sufficient for statistical analysis (Supplementary Figure S1).

**Figure 1:**
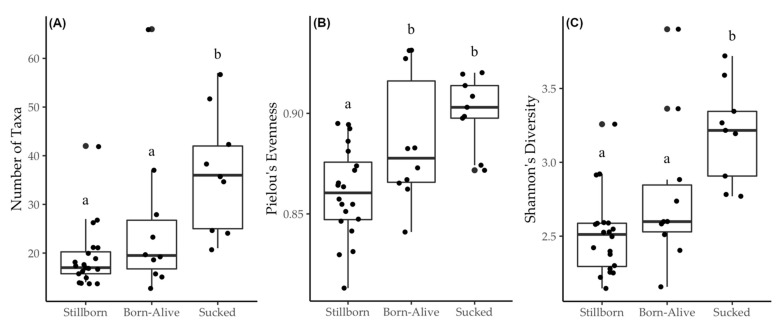
Boxplots demonstrating the change at genus level in (**A**) the number of taxa, (**B**) Pielou’s evenness, and (**C**) Shannon’s diversity for piglets that were stillborn, born-alive or had sucked. When subscripts differ, they denote a significant difference between treatments (*p* < 0.001).

**Figure 2:**
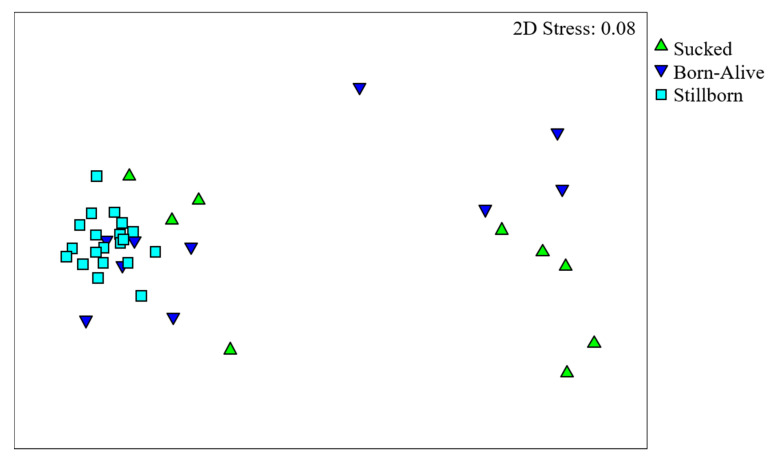
nMDS ordination showing the relationship of colonic bacterial genera from piglets that were stillborn (square), born-alive (inverted triangle) or had sucked (triangle), calculated using Bray–Curtis distances. Points on the ordination represent individual piglet samples which are positioned based on their similarity to all other samples in a two-dimensional space. The closer the samples are together in the ordination space, the more similar are their GIT microbial communities based on taxa composition and abundance.

Alpha diversity metrics showed that the number of taxa and community richness were higher in piglets that sucked when compared with stillborn and born-alive animals (*p* < 0.001; Figure 1A,C). The community evenness was lower in stillborns in comparison to born-alive (*p* < 0.001; Figure 1B), but not effected by colostrum consumption (Pielou’s evenness, *p* < 0.001; Figure 1B). Colonic bacterial genera significantly differed among piglets (ANOSIM, Global R = 0.552, *p* = 0.001), with significant pairwise difference between stillborn and those that had sucked (R = 0.804, *p* = 0.001), and stillborn and born-alive piglets (R = 0.441, *p* = 0.001). Piglets that were born-alive and either had or had not sucked did not differ (R = 0.103, *p* = 0.112). Non-metric multidimensional scaling (nMDS), on the Bray–Curtis similarity taxonomic data, generated an ordination where the closer samples are together in space, the more similar their GIT microbial communities are. This shows that the population composition of bacteria in the born-alive and sucked piglets differed from that of stillborn piglets. Furthermore, the born-alive and sucked piglets showed a highly diverse community composition, with each of these groups showing a distinct division; with some piglets grouping closer to the stillborn piglets and others not (Figure 2). At genus level, stillborn, born-alive and sucked piglets showed GIT microbial community similarities of 66%, 42% and 41%, respectively.

**Figure 3:**
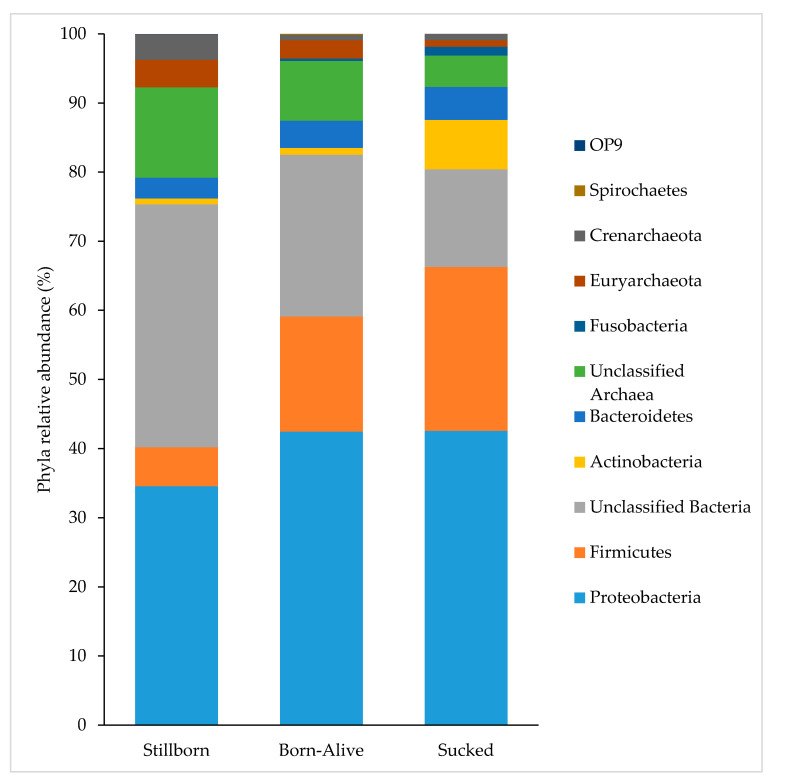
Abundance of microbial phyla present in the spiral colon of piglets that were stillborn, born-alive or those that had sucked. The bacterial phyla within the legend are arranged in the same order as they appear on the bar chart.

As shown in Figure 3, a total of 11 phyla were identified from all samples. The relative abundance of Proteobacteria remained stable as external exposure increased. Proportionally, Unclassified Archaea and Unclassified Bacteria decreased with external exposure. The abundance of Firmicutes gradually increased with external exposure while Actinobacteria, which were present in very small amounts in stillborn animals (0.85%), increased after the ingestion of milk, going from 0.97% in piglets that were born-alive to 7% in those that had sucked (Figure 3). At phylum level, the average dissimilarity between stillborn piglets and those that were born-alive and those that had sucked were 25% and 31%, respectively. The main phyla driving the differences between stillborn piglets and born-alive piglets were Firmicutes, Bacteroidetes, Unclassified Archaea, Crenarchaeota and Euryarchaeota, while those phyla driving the differences between stillborn piglets and those that had sucked were Unclassified Bacteria, Firmicutes, Actinobacteria, Unclassified Archaea, Crenarchaeota, Proteobacteria and Euryarchaeota.

**Figure 4:**
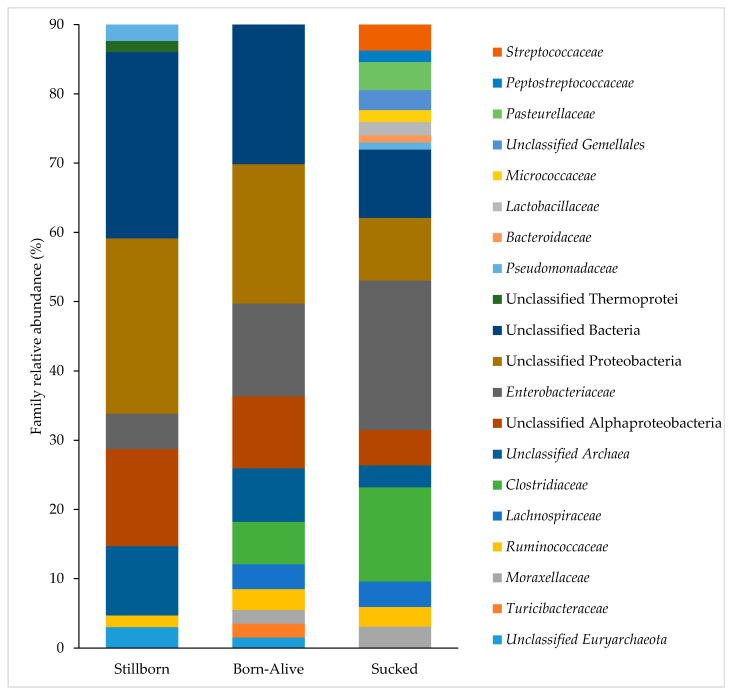
Abundance of the top 90% of microbial families present in the spiral colon of piglets that were stillborn, born-alive or those that have sucked. The bacterial families within the legend are arranged in the same order as they appear on the bar chart.

The dominant families identified in the spiral colon of piglets that were stillborn, born-alive or had sucked are shown in Figure 4. As post-natal exposure increased, the proportion of Unclassified Bacteria decreased from 27% to 21% in piglets that were born-alive and 10% in those that had sucked. Additionally, Unclassified Proteobacteria, Unclassified Alphaproteobacteria and Unclassified Archaea decreased with environmental exposure, while *Enterobacteriaceae* and *Clostridiaceae* increased with environmental exposure (Figure 4). At the family level, the average dissimilarity between piglets that were stillborn and those that were born-alive was 48%. Of the taxa that could be identified to family level, *Pseudomonadaceae* and *Bacteroidaceae* were significantly more abundant in those that were stillborn, while *Enterobacteriaceae*, *Clostridiaceae*, *Ruminococcaceae* and *Lachnospiraceae* were significantly more abundant in animals that were born-alive. When comparing stillborn piglets to animals that had sucked, the average dissimilarity at the family level was 60%. Of the taxa that could be identified to the family level, *Pseudomonadaceae* and *Ruminococcaceae* were more abundant in animals that were stillborn while *Enterobacteriaceae*, *Clostridiaceae*, *Pasteurellaceae*, *Streptococcaceae*, *Moraxellaceae*, *Lachnospiraceae*, *Lactobacillaceae*, *Micrococcaceae* and *Peptostreptococcaceae* were more abundant in animals that had sucked.

At genus level, the average dissimilarity in the spiral colon microbiota between piglets that were stillborn and those that were born-alive was 53%. Of the taxa that could be classified to the genus level and were significantly contributing to the dissimilarity, *Escherichia* and *Clostridium* were more abundant in animals that were born-alive, while *Pseudomonas*, *Bacteroides* and *Faecalibacterium* were more abundant in animals that were stillborn (Table 1). When assessing the spiral colon microbiota between piglets that were stillborn and those that had sucked, the average dissimilarity was 65%. Of the taxa that could be classified to the genus level and were significantly contributing to the dissimilarity, *Escherichia*, *Clostridium*, *Actinobacillus*, *SMB53*, *Streptococcus*, *Lactobacillus*, *Faecalibacterium* and *Roseburia* were more abundant in animals that had sucked and *Pseudomonas* were more abundant in those animals that were stillborn (Table 2). Of the taxa which could be classified to species level, *Escherichia coli*, *Clostridium perfringens* and *Prevotella copri* were more abundant in animals that were born-alive then those that were stillborn. Additionally, *E. coli*, *Clostridium celatum*, *C. perfringens*, *Lactobacillus reutueri*, *Streptococcus luteciae* and *Faecalibacterium prausnitzii* were more abundant in animals that had sucked when compared with stillborn piglets, while *Bacteroides uniformis* was more abundant in stillborns than animals that had sucked.

## 4. Discussion

It is known that the initial microorganisms colonising the GIT impacts animal health and survival, and as a result, understanding the timing of initial colonisation is crucial [1]. Additionally, as the regulation of intestinal immunity relies largely on the GIT microbiota attained by neonates in early life, piglet survival depends on ‘optimal’ microbial colonisation occurring [33]. Until recently, neonates were presumed sterile until parturition, but, studies in humans [3], mice [4] and rhesus macaques [5] have identified microorganisms within the GIT prior to parturition. To our knowledge, no other studies have investigated the intestinal microbiota of piglets prior to colostrum ingestion. The present study identified bacteria in the spiral colon of stillborn piglets, therefore the hypothesis that passive transfer of microbes would occur in the developing fetus, at least immediately prior to birth, is supported. Further, the data largely supports the second hypothesis that the composition, abundance and diversity of microbes colonising the spiral colon would increase with birth, environmental exposure and colostrum consumption.

Alpha and beta diversity metrics indicated that rapid and diverse microbial colonisation of the GIT occurred within a few hours of birth. This observation of rapid and diverse post-natal colonisation was expected and has been documented previously [13,34]. These latter authors observed the formation of dominant populations of bacteria within the first few days of life with a gradual increase in minor populations, thus increasing diversity as time progressed. In the present study, however, temperature and oxygenation of digestive tissues may have also impacted the differences observed in microbial populations in piglets that were born-alive when compared to those that were stillborn.

When comparing sample diversity, the between-sample variation was relatively low for stillborn animals, even with the majority of piglets born to different sows, while those that were born alive, regardless of whether they had sucked, showed very large between-sample variation. Additionally, a distinct split in microbial communities were observed for born-alive animals regardless of whether they had sucked, with one cluster within each group being similar to stillborn animals. The separation in microbial communities observed is likely caused by the sampling time for piglets. As some sows farrowed overnight, animals may have differed in age by as much as 12 h, therefore, it is likely that the digesta did not have enough time to reach the spiral colon prior to sampling in some animals. Additionally, there may be differences in GIT transit time between animals that had sucked and those that had not, or in the case of piglets within the sucked group, it is likely that some animals may have sucked earlier than others. Overall, this indicates that a rapid change in spiral colon microbiota occurs within the first day of life and possibly identifies a crucial time for manipulation of the microbiota through the addition of environmental substrates or diet in order to ensure it establishes in a positive state.

Interestingly, although Pielou’s evenness and taxonomic differences existed between stillborn animals and those that were born alive, regardless of whether they had sucked, stillborn and born-alive animals did not differ in Shannon’s diversity and the number of taxa. This suggests that colostrum had a major contribution to community richness but it was somewhat unexpected that animals that were born-alive did not differ in diversity and number of taxa from stillborn animals. These animals would have not only received potential exposure prior to and during parturition but would have also gained external exposure from the environment they were born into. It is likely that the amount of environmental exposure may have been insufficient to result in a significant difference in taxa number and diversity or this similarity may be a result of stillborn piglets having received some degree of microbial exposure from the vaginal fluids during parturition. However, exposure to the environment was sufficient to result in a significant change in the dominant bacteria colonising the GIT.

Stillborn piglets were used as an indicator of microbial inoculation immediately prior to birth for this experiment. It is possible that the place and time of piglet death within the reproductive tract could have influenced the colonisers observed. Studies in pigs demonstrate that the risk of stillbirth is increased by a number of factors including large litter sizes, prolonged parturition, placental detachment and umbilical cord occlusions, ruptures and breaks [19]. The majority of stillbirths are likely to occur in or above the cervix, while the vagina is the predominant location for inoculation of neonates [14]. Late-term fetuses ingest large amounts of amniotic fluid in utero and it is the ingestion of this fluid that is presumed to facilitate the colonisation of the GIT prior to birth [10]. It is possible that piglets may have ingested fluid within the cervix or vagina prior to death so, to attain the most accurate representation of microbial colonisation of the GIT in late gestation, the spiral colon of the piglet was sampled as it was assumed unlikely that any microbes ingested during parturition would not have arrived in the spiral colon within such a short time. When comparing the spiral colon microbiota of stillborn animals to the vaginal microbiota of sows from other studies it is evident that they share some dominant phyla (Firmicutes and Proteobacteria). Other phyla that are observed in dominant populations within the sow vagina are only present in very low amounts (Actinobacteria and Bacteriodetes) within stillborn piglets or are not present at all (Tenericutes) [35]. Additionally, as the variability in microbial communities between stillborn animals was low regardless of the fact that sampling time in relation to piglet death likely differed due to this death occurring during parturition. This low variability between animals suggests that the samples likely represents the environment immediately prior to birth rather than any microbes that may have been ingested during parturition. Alternately, due to the pressure exerted during parturition, the possibility of microorganisms being forced through the anus into the colon cannot be ignored. Further studies should investigate piglets prior to the onset of parturition to evaluate this. Nevertheless, if colonisation does occur during late gestation this raises the question, is there a way of influencing the microbiota of a piglet prior to parturition through the sow? It is well established within the literature that the initial microbes colonising the GIT are important for long-term health, therefore, the obvious next step would be to investigate piglets prior to parturition and whether the microbiota of a piglet can be altered in utero through sow nutritional management.

Research investigating sow microbiota identified specific bacteria present within their GIT that influence oxidative stress status and, therefore, potential stillbirth rate [36], but the taxa observed to cause this effect were not detected in the present study. Stillborn animals had consistently higher *Pseudomonadaceae*, however, information on the role it has within the gastrointestinal microbiota is not fully understood and its documentation within the GIT of pigs is limited [37]. Additionally, the present study only investigated stage II stillbirth, which are those that die during parturition, not stage I, which die prior to the onset of parturition. In the present study, there was no evidence to suggest that these bacteria are passed onto the piglet to cause stage II stillbirth, but rather it is possible that these bacteria influence internal mechanisms within the sow to cause stillbirth [36]. Alternately, the dissimilar microbial community may be a consequence of intrauterine death, however it is hoped that the sampling technique used in the present study did eliminate this possibility.

Similar to previous research, the present study demonstrated that the piglet is colonised by *Clostridiaceae* and *Enterobacteriaceae* species during the first day of postnatal life, with these being the predominant families in piglets that were born-alive and had sucked [34,38,39]. As these families were only present in small amounts within stillborn animals and increased substantially with environmental exposure and then sucking, it indicates that these taxa are most likely present within the environment and sow colostrum. This is further supported by the identification of these bacteria in piglets as prominent taxa throughout lactation [13,40]. The microbiota of sow colostrum was not evaluated, so any contribution it made remains speculative in the current study. Interestingly, high levels of archaea were observed within stillborn piglets, but these proportionally decreased as external exposure increased. Little research exists documenting the presence of archaea in piglets. Similar to the present study, Mao et al. [41] documented the presence of Euryarchaeota, which includes methanogenic archaea, and Su et al. [42] documented the presence of methanogenic archaea in the piglet from 1 day of age and saw a decrease in diversity of archaea as piglets aged. Crenarchaeota were detected in the faeces of humans, however, its mechanism of action was not completely understood [43]. While these studies identified the presence of archaea in piglets, details regarding the specific abundance and richness of archaea observed in stillborn piglets cannot be addressed by the present study as archaea-specific primers were not employed.

When examining the taxa that could be identified to the species level, it was evident that a variety of potentially pathogenic bacteria colonise very early in life and although they may not be harmful initially, if they become present in high amounts they can become pathogenic. For example, *E. coli*, *C. perfringens* and *C. celatum* were present within those animals that were born alive regardless of whether they had sucked or not, suggesting that they were likely colonised from the environment and highlighting the importance of the environmental microbiota that the piglet is born into. Similarly, a study by Chen et al. [16], identified that the microbiota from the floor, sow milk and sow nipple surface were the earliest colonisers of the piglet faecal microbiota during early lactation. These results support the importance of farrowing crate cleanliness but also highlight the possible impact of sow faeces on the initial bacteria colonising the GIT. Indeed, piglets are born onto the region of the crate where the sow urinates and defecates, however, whether this is positive or negative is yet to be elucidated as studies within our research group and others are conflicting [44]. Previous studies have also documented the high prevalence of potentially pathogenic bacteria in early life, particularly in regard to *C. perfringens* in pigs [39,45]. These latter studies also documented that as time progressed, potentially beneficial bacteria outnumbered those that were potentially pathogenic. One explanation for this is that maternal immunoglobulin A (IgA) inhibits colonisation of harmful pathogens and by day seven post-partum, IgA is the major immunoglobulin isotype identified in breast milk [46]. Additionally, the main bacteria identified in the milk of sows were lactic acid bacteria such as *Lactobacilli* and *Bifidobacterium* [45]. Therefore, it is likely that as milk consumption increases, the bacteria present within the milk dominate the bacterial shift in GIT microbiota observed as piglets age. Although previous studies demonstrated this milk microbiota shift [47], research in humans and animals suggests that it is still important for positive microbial colonisation to occur at birth in order to support long-term health and productivity [1]. Although this cannot be confirmed within the present study, further investigations into environmental bacterial exposure and the effect it has on piglet microbiota and how it impacts survival and productivity are warranted.

## 5. Conclusions

To our knowledge, this is the first study to characterise the microbiota of piglets that are stillborn or born-alive prior to sucking, facilitating the identification of bacteria that colonise the spiral colon immediately prior to birth and the initial colonisers following parturition. The results suggest that the colonisation of the GIT of a piglet occurs immediately prior to birth and that following parturition, rapid and diverse colonisation of the GIT occurs, with this colonisation being driven by the environment and the consumption of colostrum. Further investigation into the role the vaginal and environmental microbiota have on these initial colonisers is needed in order to understand the origin of the potentially pathogenic bacteria observed in the current study. Indeed, a potential limitation of this study is that piglets were not sampled pre-partum. This could be addressed in the future by sampling in late gestation. Investigation of the amniotic and placental microbiota of pre-term piglets would help to understand the accuracy surrounding the stillborn samples attained in the present study and to determine the origin of the bacteria and archaea colonising. This will aid in determining the potential to influence the gastrointestinal microbiota of the developing fetus.

## Figures and Tables

**Table 1:** Taxa contributing significantly (average dissimilarity/standard deviation > 1) to the dissimilarity between born-alive and stillborn piglets as determined by SIMPER analysis at the genus level.

	Born-Alive	Stillborn	
Genus	Average Abundance	Average Abundance	%
Unclassified Archaea	0.67	1.03	2.15
Unclassified Thermoprotei	0.18	0.30	1.85
Unclassified Crenarchaeota	0.07	0.19	1.20
Unclassified Euryarchaeota	0.19	0.35	1.11
*Clostridium*	0.98	0.09	5.71
*Faecalibacterium*	0.10	0.11	0.79
Unclassified Alphaproteobacteria	1.00	1.44	2.91
Unclassified Rickettsiales	0.14	0.21	1.06
*Escherichia*	1.59	0.67	7.11
*Pseudomonas*	0.19	0.53	2.96
*Bacteroides*	0.14	0.15	0.82

Overall average dissimilarity between treatments was 53%. Only taxa that were significantly different between treatments are shown in this table. % represents the percentage contribution for these bacteria.

**Table 2:** Taxa contributing significantly (average dissimilarity/standard deviation > 1) to the dissimilarity between piglets that had sucked and those that were stillborn as determined by SIMPER analysis at the genus level.

	Sucked	Stillborn	
Genus	Average Abundance	Average Abundance	%
Unclassified Archaea	0.54	1.03	2.17
Unclassified Crenarchaeota	0.13	0.19	0.76
Unclassified Thermoprotei	0.10	0.30	1.16
Unclassified Euryarchaeota	0.16	0.35	0.92
Unclassified Bacteria	1.47	2.66	5.11
Unclassified Gemellales	0.45	0.03	1.85
*Lactobacillus*	0.39	0.04	1.53
*Streptococcus*	0.60	0.10	2.45
Unclassified *Clostridiaceae*	0.42	0.00	1.73
*Clostridium*	1.25	0.09	5.07
*SMB53*	0.74	0.04	3.19
Unclassified *Peptostreptococcaceae*	0.31	0.00	1.29
Unclassified *Lachnospiraceae*	0.16	0.02	0.69
*Roseburia*	0.11	0.06	0.45
*Faecalibacterium*	0.16	0.11	0.58
Unclassified Proteobacteria	1.39	2.50	4.78
Unclassified Alphaproteobacteria	0.80	1.44	2.89
Unclassified Rickettsiales	0.07	0.21	0.82
*Escherichia*	2.28	0.67	7.20
*Actinobacillus*	0.83	0.00	3.37
*Pseudomonas*	0.30	0.53	2.01
Unclassified *S24-7*	0.13	0.02	0.55

Overall average dissimilarity between treatments is 65%. Only taxa that were significantly different between treatments are shown in this table. % represents the percentage contribution for these bacteria.

## Data Availability

All data generated or analysed during this study are included in this published article or can be found in the Sequence Read Archives of the NCBI database under accession number PRJNA677620.

## Supplementary Materials

The following are available online at https://www.mdpi.com/article/10.3390/life11040312/s1, Supplementary Figure S1: Species accumulation curves and richness indices of the bacterial communities in the spiral colon of piglets in the stillborn, born-alive and sucked groups.
