# Supplementary material for: Characterisation of Early Microbial Colonisers within the Spiral Colon of Pre- and Post-Natal Piglets
Source: Life (Basel). 2021 Apr 2;11(4):312. doi: 10.3390/life11040312 (PMC8066224; doi:10.3390/life11040312)
Supplement: Supplementary file 1 [file life-11-00312-s001.pdf]

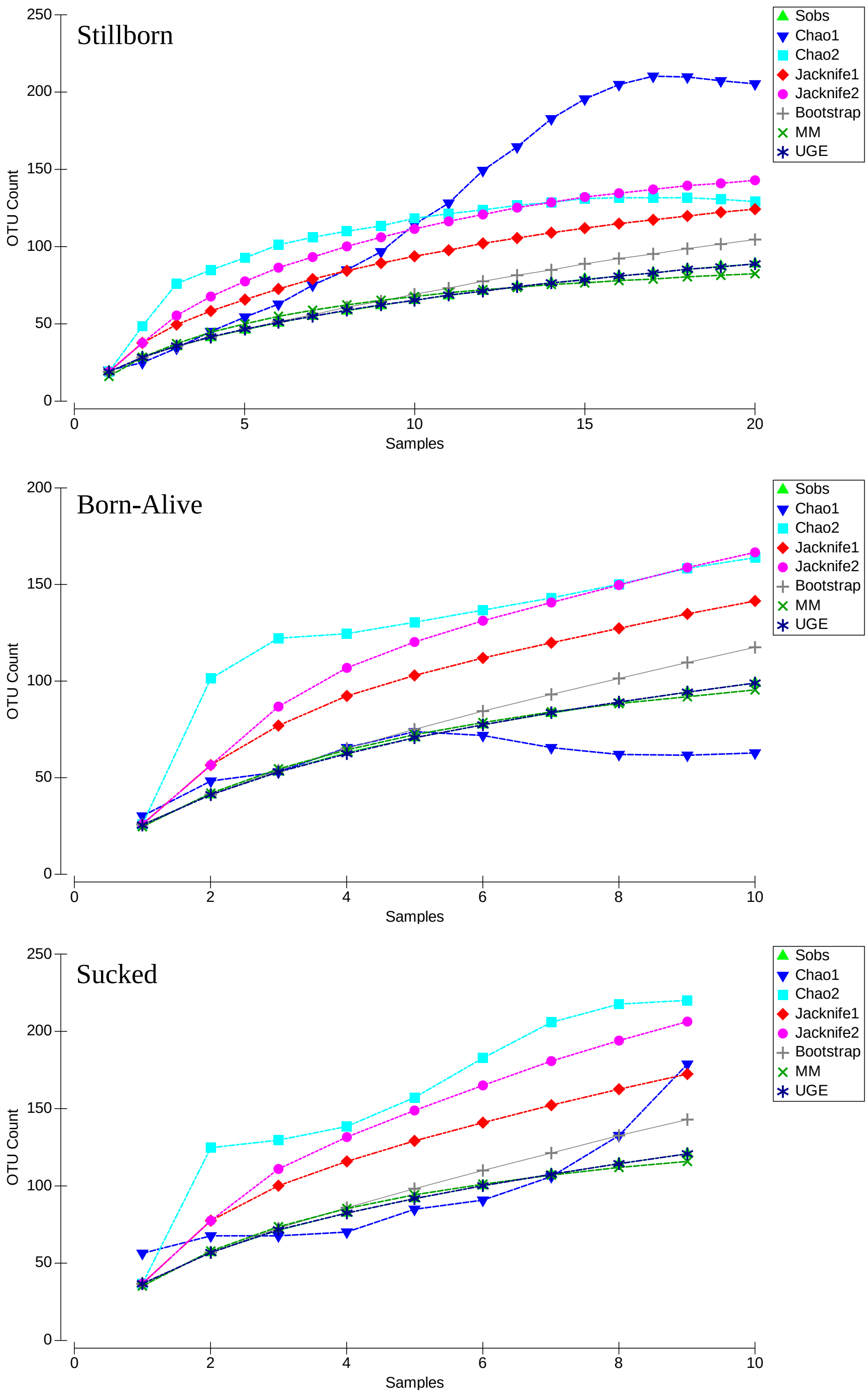

Supplementary Figure 1. Species accumulation curves and richness indices of the bacterial communities in the spiral colon of piglets in the stillborn, born-alive and sucked groups. Green triangle represent the observed accumulated species richness (Sobs). Blue upside-down triangle and blue square represent the abundance-based estimator of species richness (Chao). Red diamond and pink circle represent the absence or presence-based estimator of species richness (Jacknife). Grey plus sign represents an estimator of true richness (Bootstrap). Green

“X” sign representing Michaelis-Menten (MM), a parametric approach to richness and the blue star represents true regional richness given by the Ugland-Gray-Elligsen (UGE) index.
